# *SPP2* Mutations Cause Autosomal Dominant Retinitis Pigmentosa

**DOI:** 10.1038/srep14867

**Published:** 2015-10-13

**Authors:** Yuan Liu, Xue Chen, Qihua Xu, Xiang Gao, Pancy O. S. Tam, Kanxing Zhao, Xiumei Zhang, Li Jia Chen, Wenshuang Jia, Qingshun Zhao, Douglas Vollrath, Chi Pui Pang, Chen Zhao

**Affiliations:** 1 Department of Ophthalmology, The First Affiliated Hospital of Nanjing Medical University and State Key Laboratory of Reproductive Medicine, Nanjing Medical University, Nanjing 210029, China; 2 Department of Ophthalmology, The Affiliated Jiangyin Hospital of Southeast University Medical College, Jiangyin, China; 3 Department of Ophthalmology, School of Medicine, Henan Polytechnic University, Henan 454150, China; 4 Department of Ophthalmology & Visual Sciences, The Chinese University of Hong Kong, Hong Kong; 5 Tianjin Medical University, Tianjin Eye Hospital, Tianjin Key Laboratory of Ophthalmology and Visual Science, Tianjin 300040, China; 6 Model Animal Research Center, MOE Key Laboratory of Model Animal for Disease Study, Nanjing University, Nanjing 210061, China; 7 Department of Genetics, Stanford University School of Medicine, CA 94305, USA; 8 State Key Laboratory of Ophthalmology, Zhongshan Ophthalmic Center, Sun Yat Sen University, Guangzhou, China

## Abstract

Retinitis pigmentosa (RP) shows progressive loss of photoreceptors involved with heterogeneous genetic background. Here, by exome sequencing and linkage analysis on a Chinese family with autosomal dominant RP, we identified a putative pathogenic variant, p.Gly97Arg, in the gene *SPP2*, of which expression was detected in multiple tissues including retina. The p.Gly97Arg was absent in 800 ethnically matched chromosomes and 1400 in-house exome dataset, and was located in the first of the two highly conserved disulfide bonded loop of secreted phosphoprotein 2 (Spp-24) encoded by *SPP2*. Overexpression of p.Gly97Arg and another signal peptide mutation, p.Gly29Asp, caused cellular retention of both endogenous wild type and exogenous mutants *in vitro*, and primarily affected rod photoreceptors in zebrafish mimicking cardinal feature of RP. Taken together, our data indicate that the two mutations of *SPP2* have dominant negative effects and cellular accumulation of Spp-24 might be particularly toxic to photoreceptors and/or retinal pigment epithelium. *SPP2* has a new role in retinal degeneration.

Retinitis pigmentosa (RP, MIM 268000) is the most common form of inherited retinal dystrophies (IRDs) and affect over one million individuals globally^1^. During the disease course, rod photoreceptors and/or retinal pigment epithelium (RPE) cells are primarily affected by most mutations leading to night blindness and gradual constriction of visual fields (VFs). Degeneration of cone photoreceptors is generally secondary to rods degeneration^2^,^3^, and therefore may ultimately cause the loss of central vision.

RP exhibits great genetic heterogeneity involving 64 disease-causing genes, of which 24 genes are implicated in autosomal dominant RP (adRP) (www.RetNet.org). Proteins encoded by those genes are implicated in multiple functions, including phototransduction, visual cycle, homeostasis of photoreceptors, metabolism and phagocytosis of RPE cells, and some fundamental cellular activities such as pre-mRNA splicing^4^. Some proteins are retina specific, while others are widely, or even ubiquitously, expressed. However, mutations in all identified RP genes can only account for 50% to 60% of all cases, leaving the heritability in nearly 40% of RP patients unknown^5^,^6^,^7^,^8^.

The *SPP2* gene (MIM 602637; NG_008668.1) spans approximately 27 kb at chromosome 2q37.1, contains 8 exons, and encodes the secreted phosphoprotein 24 (Spp-24; NP_008875.1), which is considered as a member of the cystatin superfamily^9^. Spp-24 consists of 211 amino acids including a signal peptide (the first 29 residues), a cystatin-like domain, in which two highly conserved disulfide bonds reside (Cys92 - Cys103, and Cys116 - Cys134), and a variable C-terminal region^9^. While the human *SPP2* is a new and relatively poor characterized gene, the cystatin-like domain of the mature bovine spp-24, after the cleavage of the signal peptide, was proposed to form a tertiary structure similar to cystatins functioning as an inhibitor of the thiol proteases such as cathepsins^10^,^11^.

Herein, using whole exome sequencing (WES), we identified a heterozygous missense variant in the gene *SPP2*, p.Gly97Arg, in a four-generation Chinese family with RP. The p.Gly97Arg is located within the first disulfide bonded loop in the cystatin-like domain of Spp-24. To determine the pathogenicity of *SPP2* p.Gly97Arg, we characterized the phenotypes in cells and zebrafish overexpressed with wild type Spp-24 (Spp-24^WT^), mutant Spp-24 carrying p.Gly97Arg (Spp-24^Gly97Arg^) and an artificial mutation, p.Gly29Asp (Spp-24^Gly29Asp^), a presumable positive control mutant predicted to affect the signal peptide cleavage and protein secretion. We found that overexpression of Spp-24^Gly97Arg^ and Spp-24^Gly29Asp^, but not Spp-24^WT^, caused cellular retention of exogenous Spp-24 in HEK 293T cells and initiated demorphogenesis of rod photoreceptors in zebrafish larvae. Altogether, we demonstrate that *SPP2* is a new disease-causing gene for RP.

## Results

### Clinical evaluations

**Figure 1:**
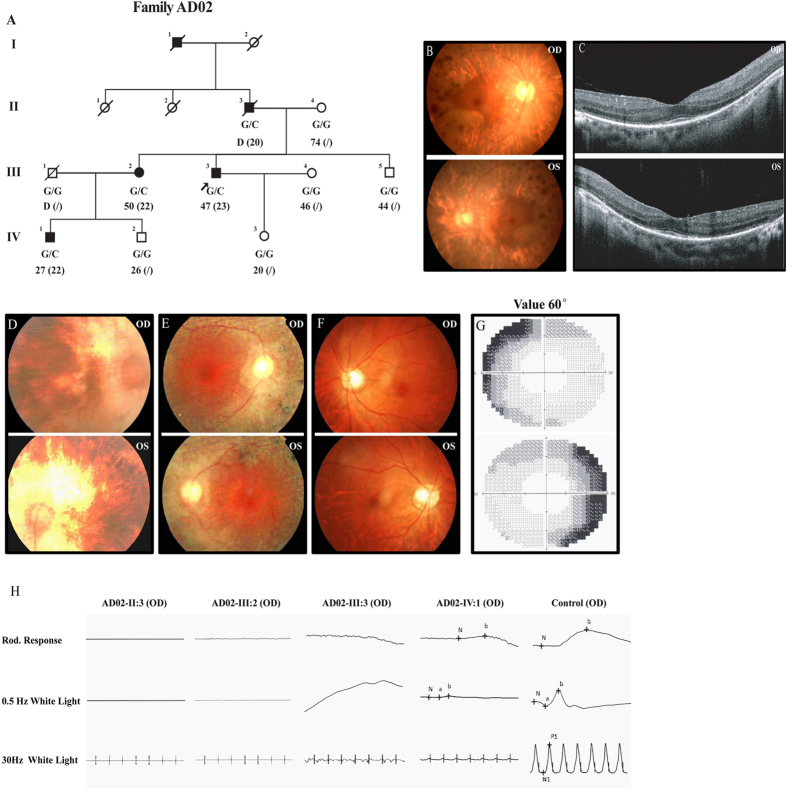
Detailed clinical evaluations of patients from family AD02. (**A**) The pedigree of family AD02 indicates a dominant inheritance pattern of four generations. Genotypes, current and onset ages of RP (inside parentheses) are shown below the pedigree symbols. (**B**,**D**–**F**) Fundus photos demonstrate typical RP changes including waxy optic disks, artery attenuations, pigment deposits, and macular degeneration, in both eyes of patient AD02-III:3 (**B**), AD02-II:3 (**D**) and patient AD02-III:2 (**E**), while the fundus of patient AD02-IV:1 was normal (**F**). (**C**) Macular degeneration is also indicated by OCT examinations, which reveal attenuated ONL and RPE with complete loss of OS and IS. (**G**) Peripheral vision loss is revealed by automated visual field examination of patient AD02-IV:1. (**H**) Scotopic and photopic ERG responses of patients AD02-II:3, III:2, and III:3 are undetectable, while are significantly reduced for patient AD02-IV:1. ERG responses of a negative control are also presented.

The proband of family AD02 (Fig. 1A), AD02-III:3, was first referred to ophthalmic examinations at age 23 due to poor night vision. At his last visit (age 47), his VF was constricted to less than 15 degrees in both eyes, while his central vision was relatively preserved (Table 1). Funduscopy showed bilateral RP characteristics including waxy pallor of the optic discs, retinal atrophy with macular region relatively preserved, and pigmental deposits in midperiphery (Fig. 1B). Optical coherence tomography (OCT) demonstrated moderate degeneration of the outer nuclear layer (ONL) in macular region with significant loss of inner/outer segments (IS/OS) (Fig. 1C). His rod photoreceptor responses were nearly diminished as demonstrated by full-field electroretinogram (ERG) (Fig. 1H). Patients AD02-II:3 and AD02-III:2 noticed decreased night vision at 20 and 22-year-old respectively. Patients AD02-II:3 recently deceased, but at his last visit (age 77), he had developed server bilateral cataract with poor best-corrected visual acuities (BCVAs). Patient AD02-III:2 still had functional BCVAs and preserved maculae at her latest visit (age 50). Their latest clinical documentations including BCVAs, VF, fundus change and full-field ERG were detailed in Table 1 and Fig. 1D,E,H. Patient AD02-IV:1 reported decreased night vision at age 22, but his fundus showed no remarkable change with normal BCVAs at the last visit (27-year-old). However, significantly reduced ERG responses and moderately reduced peripheral VF were detected (Fig. 1F–H and Table 1).

### Identification of putative variant in *SPP2* gene

**Figure 2:**
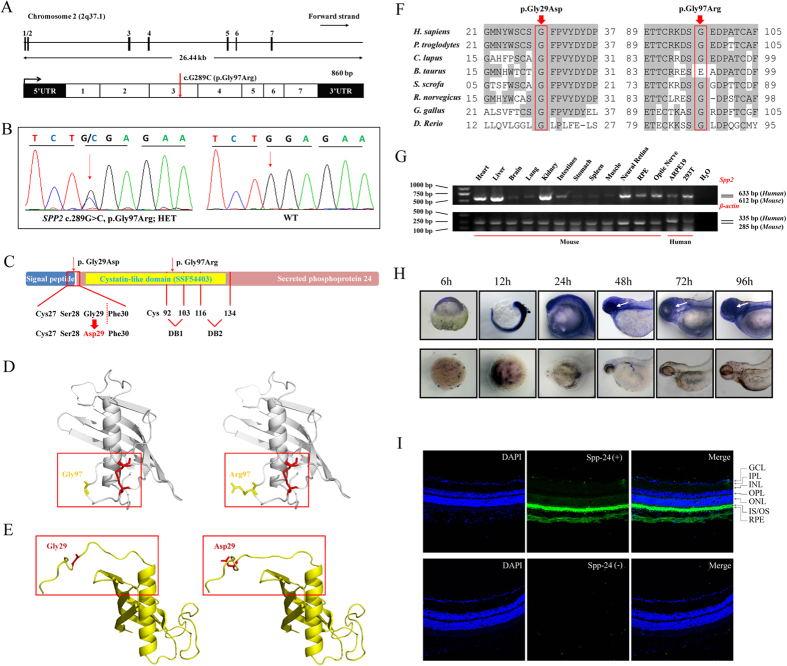
Genetic analyses of the *SPP2* variants, and expression profiling of Spp-24. (**A**) *SPP2* gene spanning 26.44 kb on chromosome 2q37.1 contains 7 exons (upper panel). The identified heterozygous variant, c.G289C (p.Gly97Arg), was located within exon 3 (below panel). (**B**) Sequencing chromatogram of patient AD02-III:3 shows the c.289G>C substitution. WT sequence is also shown. (**C**) Schematic representation of the relative linear location of the two *SPP2* mutations in context of Spp-24 protein structure. The p.Gly97Arg is located in the cystatin-like domain, and the artificial mutation, p.Gly29Asp, is located at the last residue of signal peptide. The red dotted line denotes the cleavage site of the signal peptide. The two internal disulfide bonds are indicated by DB1 and DB2 respectively. (**D**–**E**) Structural modeling of Spp-24. The mutated residue 97 is located within the first disulfide bond loop of Spp-24 (**D**, cys92-103 disulfide bond is denoted by red color). The other substitution p.Gly29Asp would lead to the distortion of the signal peptide cleavage site in the mutant when compared with the WT monomer (**E**). (**F**) Conservation analyses of the mutated residues 29 and 97 of Spp-24 in multiple species, including human (*H. sapiens*), chimpanzees (*P. troglodytes*), dogs (*C. lupus*), cows (*B. taurus*), pigs (*S. scrofa*), rats (*R. norvegicus*), chickens (*G. gallus*), and zebrafish (*D. rerio*). Conserved residues are shaded. (**G**) Expression of *SPP2* in multiple murine tissues and human cell lines, namely ARPE19 and HEK 293T (upper panel). Expression of β-actin serves as internal control (lower panel). (**H**) Zebrafish whole mount *in situ* hybridization reveals the ubiquitously expression of the *spp2* transcript throughout the development of zebrafish. A relatively higher expression in the eye wall after 48 hours post fertilization is indicated by white arrows. (**I**) Immunostaining for spp-24 (green) on murine retinal frozen section. Abundant reactivity of spp-24 was detected in the inner/outer segments (IS/OS) and retinal pigment epithelium (RPE) layers, and moderate staining was also found in sclera, choroidal vessels and ganglion cells (GC) (upper panel). Sections incubated with secondary antibody alone serve as negative control (lower panel). Scale bar: 100 μm.

The pedigree of the family AD02 indicated a likely autosomal dominant inheritance mode because of the presence of patients in each generation with roughly 50% occurrence and the male-to-male transmission (Fig. 1). To determine the RP causative mutation for this family, targeted next-generation sequencing was initially conducted on 3 individuals, including patient AD02-II:3, patient AD02-IV:1, and the unaffected AD02-III:5, to detect potential disease causative mutation in identified RP relevant genes. No potential disease causative mutation was revealed. WES was further performed on these 3 individuals. The mean depths of targeted region yielded by WES were 66.72-, 82.10-, and 74.60-fold for individuals AD02-II:3, AD02-IV:1, and AD02-III:5, respectively. The detailed WES data were summarized in Supplemental Table 1. After initial screening, a total of 37 single nucleotide variations (SNVs) were shared by the two patients (AD02-II:3 and AD02-IV:1) but not the unaffected member (AD02-III:5), and were absent in 5 SNP databases. Further co-segregation analysis of these SNVs among all family members recruited was performed by Sanger sequencing with their primers detailed in Supplemental Table 2. Among them, four SNVs were found to be false positive, five did not fit with the autosomal dominant inheritance mode, and another 27 SNVs did not co-segregate with the disease phenotype in the family. Only one heterozygous SNV, c.G289C in the exon 3 of *SPP2* gene (Fig. 2A–C), was completely co-segregated with RP phenotype of this family. It was further found to be absent in 800 unrelated ethnically matched alleles and the in-house exome sequencing database derived from 1400 Chinese unrelated to RP. The variant c.G289C would result in an amino acid substitution at position 97 of Spp-24 (p.Gly97Arg), a switch from a neutral amino acid, glycine, to an alkaline amino acid, arginine. We next directly sequenced all 7 coding exons of *SPP2* gene in patients AD02-II:3 and AD02-IV:1. No other pathogenic variant in *SPP2* was detected.

### Comparative and structural analyses of *SPP2* variants

To predict the potential pathogenic impact of p.Gly97Arg on protein level, we carried out comparative and structural analyses. Multiple orthologous sequence alignment (MSA) revealed that p.Gly97Arg was conserved among all species tested except for cow (Fig. 2F). Similarly, p.Gly97Arg was predicted to be possibly damaging by 3 out of the 4 programs (Table 2).

We next modeled the crystal structure of wild type and mutant Spp-24 protein with p.Gly97Arg using a template of human cystatin F [Protein Data Base (PDB) ID: 2CH9]^12^, a member of type II cystatins^12^ that shows the highest structural similarity to Spp-24 according to the analysis from SWISS MODEL. The predicted structure of Spp-24 was covered from residues number 22 to 136 and shared 28% homology to the template protein in sequence. Variant p.Gly97Arg was located in the cystatin-like domain within the first of the two disulfide bonded loops of Spp-24 (Fig. 2D,E), which are key motifs shared by all Spp-24 proteins and type II cystatins^9^,^12^. Of note, the p.Gly97Arg represented a substitution from a relatively hydrophobic and conformational flexible residue to a highly hydrophilic and conformational rigid residue.

### Expression of the *SPP2* gene

We first analyzed the expression of *SPP2* gene in a panel of murine tissues, and human HEK 293T and ARPE19 cells using reverse transcriptase polymerase chain reaction (RT-PCR) analysis. Expression of *Spp2* was clearly detected in murine liver, kidney, heart, RPE and neural retina (Fig. 2G), and was also detectable in several other tissues and the two tested human cell lines, suggesting a wide expression of this gene. We also determined the expression of *Spp2* in zebrafish at different stages by whole mount *in situ* hybridization, and similarly, the *spp2* transcript was ubiquitously expressed during development of zebrafish and was significantly expressed in the eye wall after 48 hours post fertilization (Fig. 2H).

To further localize the spp-24 protein in ocular tissues, we conducted immunostaining for spp-24 in murine eye sections. Reactivity of the Spp-24 protein was most abundant in IS/OS and RPE, and was also detectable in the ONL, ganglion cells (GC), choroidal vessels and sclera (Fig. 2I). In the negative control sections incubated with second antibody only, no non-specific staining was found (Fig. 2I).

### *SPP2* variants cause Spp-24 cellular retention in the endoplasmic reticulum

We hypothesized that the p.Gly97Arg may affect the cellular localization and secretion of Spp-24. To this end, we transiently expressed the C-terminally Flag-tagged fusion protein of Spp-24^WT^, Spp-24^Gly97Arg^, and Spp-24^Gly29Asp^ in HEK 293T cells respectively. The plasmid Spp-24^Gly29Asp^ carries an artificial mutation, p.Gly29Asp, which serves as a potential positive control for the secretory defect of Spp-24. The p.Gly29Asp affects an absolutely conserved site according to MSA analysis (Fig. 2C), and is predicted to be deleterious by all online prediction programs applied (Table 2). More importantly, the p.Gly29Asp is located at the last residue of the signal peptide. As indicated by SignalP program, the Y score peaks at the residue Phe30 of Spp-24^WT^, and at the residue Ser28 of the mutant Spp-24^Gly29Asp^. In addition, modeling of Spp-24 revealed that the p.Gly29Asp altered the orientation of the corresponding residue (Fig. 2E). Thus, p.Gly29Asp highly affects the conformation and the cleavage of the signal peptide leading to secretory defect of Spp-24.

**Figure 3:**
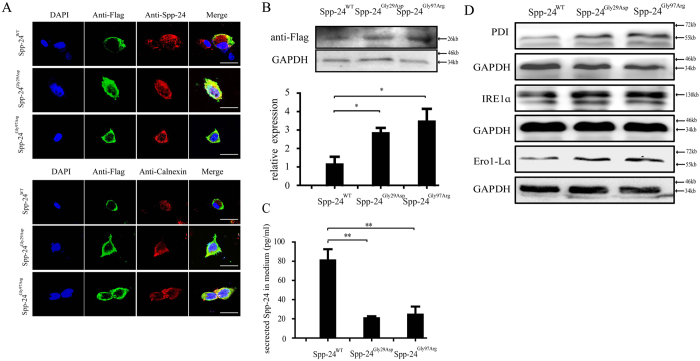
Analyses of the cellular processing of wild type and mutant Spp-24 in transfected HEK 293T cells. (**A**) Immunofluorescence staining of HEK 293T cells transiently transfected with Flag-tagged wild type secreted phosphoprotein 2 (Spp-24^WT^), mutant Spp-24 carrying p.Gly29Asp (Spp-24^Gly29Asp^) and p.Gly97Arg (Spp-24^Gly97Arg^), respectively. Cell nuclei are presented using DAPI. Images are also shown as anti-Flag (detecting exogenous Flag-tagged Spp-24), anti-Spp-24 (detecting both endogenous and exogenous Spp-24), anti-Calnexin (endoplasmic reticulum), and merged formats. Scale bar: 20 μm. (**B**) Immunoblot of Flag on cellular protein extracts from HEK 293T cells transfected with Spp-24^WT^, Spp-24^Gly29Asp^ and Spp-24^Gly97Arg^. The glyceraldehyde-3-phosphate dehydrogenase (GAPDH) serves as internal control. The intracellular amount of exogenous Spp-24 as indicated by the density of bands for Flag was quantified and normalized to internal control (indicated by bar graph at bottom, Spp-24^WT^ VS Spp-24^Gly29Asp^, P = 0.0463; Spp-24^WT^ VS Spp-24^Gly97Arg^, P = 0.0313). Error bars represent standard deviation (STDV) from biological triplicates. (**C**) Enzyme-linked immuno sorbent assay (ELISA) is performed to determine the amount of Spp-24 in the culture medium of the cells transfected with Spp-24^WT^, Spp-24^Gly29Asp^ and Spp-24^Gly97Arg^, respectively (Spp-24^Gly29Asp^ VS Spp-24^WT^, P = 0.0024; Spp-24^Gly97Arg^ VS Spp-24^WT^, P = 0.003). Error bars represent STDV from biological triplicates. *P < 0.05, **P < 0.01. (**D**) Immunoblots of PDI, IRE1α, and Ero1-Lα on cellular protein extracts from HEK 293T cells transfected with Spp-24^WT^, Spp-24^Gly29Asp^ and Spp-24^Gly97Arg^. The GAPDH serves as internal control.

The subcellular localizations of the exogenous Spp-24 were first determined by immunofluorescent staining of Flag (Fig. 3A). As expected, for cells transfected with Spp-24^WT^, the exogenous Spp-24^WT^ is found mainly localized to the cell membrane, while the endogenous Spp24 protein shows a uniform distribution in the transfected cells (Fig. 3A). However, for cells overexpressing mutant Spp-24^Gly29Asp^ or Spp-24^Gly97Arg^, the exogenous Flag-tagged mutant Spp-24 showed extensive co-localization with the endogenous Spp-24 protein in the cytoplasm and perinucleus region, indication a potential interaction between the mutant and the wild type Spp-24 proteins (Fig. 3A). The membranous Spp-24^Gly97Arg^ and Spp-24^Gly29Asp^ was much less distinct when compared to Spp-24^WT^ as indicated by the staining of Flag (Fig. 3A). In addition, the exogenous mutant Spp-24 protein also showed colocalization with the endoplasmic reticulum (ER), indicating retention of the Spp-24 proteins in ER (Fig. 3A).

We further assessed the cellular protein amount of exogenous Spp-24 in HEK 293T cells by immunoblot of Spp-24. Consistently, we found that the cellular Spp-24^Gly29Asp^ and Spp-24^Gly97Arg^ were increased when compared with Spp-24^WT^ (Fig. 3B). As Spp-24 is a secreted protein, we next applied ELISA to determine the secreted Spp-24 in the culture medium of the cells transfected with each of the three different Spp-24 plasmids. Consistent to the immunostaining result, the secreted Spp-24 was found significantly less in the cells transfected with Spp-24^Gly29Asp^ or Spp-24^Gly97Arg^ when compared with cells overexpressing Spp-24^WT^ (Fig. 3C). Immunoblotting also indicated that expressions of markers relevant to ER-stress, including protein disulfide isomerase (PDI), IRE1α, and ER protein endoplasmic oxidoreductin-1-Lα (Ero1-Lα), were elevated in cells overexpressing Spp-24^Gly29Asp^ or Spp-24^Gly97Arg^ when compared with cells overexpressing Spp-24^WT^. Taken together, our cellular studies thus indicated that the RP-associated variant, p.Gly97Arg, and the artificial variant, p.Gly29Asp, have similar pathogenic effect causing cellular retention of both endogenous and exogenous Spp-24 in ER, which further induced ER stress. Overexpression of the two mutants in zebrafish could result in retinal phenotypes via dominant-negative effect leading to loss-of-function of the protein.

### Overexpressing mutant Spp-24 initiates defects in rod photoreceptors and increases systemic deformity in zebrafish

**Figure 4:**
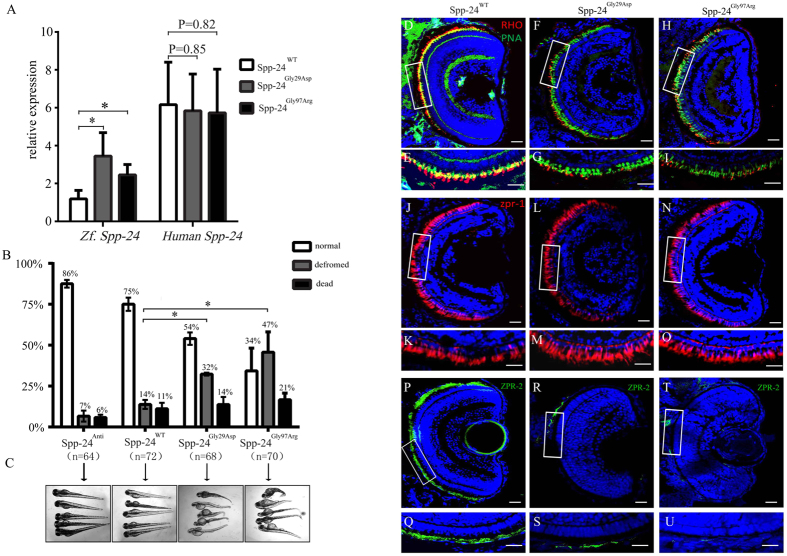
Deleterious effects identified in zebrafish overexpressing Spp-24 mutants. (**A**) Expressions of endogenous *spp2* (Zf.) and exogenous *SPP2* (Human) in zebrafish at 2 days post fertilization (dpf) after injection of Spp-24^WT^, Spp-24^Gly29Asp^ and Spp-24^Gly97Arg^ were determined by Q-PCR and were relative to the expression of spp2 in uninjected larvae. (Spp-24^Gly29Asp^ VS Spp-24^WT^, P = 0.0463; Spp-24^Gly97Arg^ VS Spp-24^WT^, P = 0.0379). (**B**) Quantification of normal, deformed, and dead zebrafish injected with different mRNAs from 2 to 4 dpf. n: total numbers of injected zebrafish from triple experiments. (Spp-24^Gly29Asp^ VS Spp-24^WT^, P = 0.0142; Spp-24^Gly97Arg^ VS Spp-24^WT^, P = 0.0105). (**C**) Morphological changes in zebrafish injected with different mRNAs at 4 dpf. Most zebrafish injected with Spp-24^Anti^ and Spp-24^WT^ is relatively normal, while significant systemic deformations are revealed in groups injected with Spp-24^Gly29Asp^ and Spp-24^Gly97Arg^. (**D**–**U**) Immunostaining of rhodopsin (**D**–**I**), peanut agglutinin (PNA) lectin (**D**–**I**), ZPR-1 (**J**–**O**), and ZPR-2 (**P**–**U**) on retinal frozen sections of zebrafish at 4 dpf from the four groups, including groups injected with Spp-24^WT^, Spp-24^Gly29Asp^ and Spp-24^Gly97Arg^. Robust staining of rhodopsin is found in the rod IS/OS and cone OS layers in Spp-24^WT^ -injected fish, respectively (**D**–**E**). Reactivity of rhodopsin (**F**–**I**) and ZPR-2 (**Q**–**U**) are decreased in Spp-24^Gly29Asp^ and Spp-24^Gly97Arg^-injected zebrafish, while reactivities of ZPR-1 and PNA was clearly detected in the IS/OS layer of all zebrafish studied (**D**–**O**). The boxed areas in (**D**,**F**,**H**,**J**,**L**,**N**,**P**,**R**,**T**) were shown in higher magnification in (**E**,**G**,**I**,**K**,**M**,**O**,**Q**,**S**,**U**). Scale bar: 20 μm.

To further investigate the pathogenicity of the two Spp-24 mutations *in vivo*, we used the zebrafish model. Embryos were divided into four groups and injected with 100 ng (1 nl) of distinct human mRNA including *SPP2* antisense mRNA (Spp-24^Anti^, n = 64) as negative control, wild type *SPP2* mRNA (Spp-24^WT^, n = 72), and two mutant *SPP2* mRNAs (Spp-24^Gly29Asp^, n = 68; Spp-24^Gly97Arg^, n = 70). Quantitative PCR (Q-PCR) showed similar levels of expressions of exogenous *SPP2* at 2 days post fertilization (dpf) among the three groups injected with Spp-24^WT^, Spp-24^Gly29Asp^ and Spp-24^Gly97Arg^ (Fig. 4A). However, the expression of endogenous *spp2* was significantly elevated by the injection of Spp-24^Gly29Asp^ (3.45 ± 1.49 fold) and Spp-24^Gly97Arg^ (2.45 ± 0.82 fold), when compared with Spp-24^WT^-injected larvae and non-injected group (default as 1 fold). Presumably there was a compensatory response of endogenous *spp2* to the over-expressed mutant human *SPP2* (Fig. 4A). Because we found that *SPP2* was widely expressed, we next compared the systemic effects relevant to the injection of different *SPP2* mRNAs. At 4 dpf, we observed dramatic systemic deformation in substantial number of larvae overexpressing Spp-24^Gly29Asp^ (~32%) and Spp-24^Gly97Arg^ (~47%), including brain malformation, cardiac edema, short trunks, and curved body axis. The proportions of zebrafish with deformity in the two groups were significantly increased when compared to the Spp-24^WT^-injected (~14%) and Spp-24^Anti^-injected (~7%) zebrafish (Fig. 4B,C). The overexpression of Spp-24^Gly97Arg^ has also caused significantly increased death rate (~21%) when compared to the Spp-24^WT^-injected (~11%) and the Spp-24^Anti^-injected (~6%) groups (Fig. 4B). Injection of Spp-24^WT^, compared to injection of Spp-24^Anti^, has also slightly increased the rates of deformity and death, presumably due to toxicity of overexpressed exogenous protein as we previously found in other zebrafish models^13^.

To further correlate the SPP2 mutations with retinal phenotypes, we investigated the integrity of retina in those zebrafish less affected by overexpression of the two mutant proteins, under the assumption that the two mutations primarily affect retinal cells. To this end, zebrafish with a normal systematic appearance were randomly selected from the three groups injected with Spp-24^WT^, Spp-24^Gly29Asp^ and Spp-24^Gly97Arg^ at 4 dpf. As expected, immunofluorescent staining of rhodopsin, a specific marker for rod photoreceptors, revealed greatly decreased expression of rhodopsin in the eye sections of the majority of zebrafish injected with Spp-24^Gly29Asp^ (n = 14 out of 25) and Spp-24^Gly97Arg^ (n = 13 out of 20), whereas abundant specific staining of rhodopsin was constantly observed in the inner/outer segment (IS/OS) layer of all larvae injected with Spp-24^WT^ (n = 20) (Fig. 4D–I). We next determined the morphogenesis of cone photoreceptors in these models using an antibody against ZPR-1, a cone photoreceptor-specific marker, and peanut agglutinin (PNA) lectin, labeling the cone OS (Fig. 4D–I). PNA staining, similar to ZPR-1 staining, was also clearly detected in the IS/OS layer of all zebrafish studied (Fig. 4J–O). Similar to results observed in rhodopsin staining, immunofluorescent staining of ZPR-2, a RPE specific marker, showed significant reduction in intensity of zebrafish injected with Spp-24^Gly29Asp^ and Spp-24^Gly97Arg^ when compared with those overexpressing Spp-24^WT^ (Fig. 4P–U).

## Discussion

In the *SPP2* genes family, bovine spp-24 was the first characterized and suggested to be involved in bone metabolism and fetutin-mineral complex^14^. Subsequently, human Spp-24 was defined with similar structure and was detected in kidney, liver and plasma^9^. In the present study, we identified a heterozygous missense mutation in *SPP2*, p.Gly97Arg, which was associated with adRP in a four-generation Chinese family. Moreover, we found that *SPP2* was detectable in retina at both transcript and protein level, and overexpression of mutant Spp-24 caused primary defects in rod photoreceptors mimicking the cardinal feature of RP. Altogether, our data indicate a new role of Spp-24 in the pathogenesis of retinal degeneration.

The Spp-24 proteins contain four absolutely conserved cysteines to form 2 internal disulfide bonded loops, which are also key motif of type II cystatins^9^,^12^. The second disulfide bonded loop in bovine spp-24 is a highly conserved bone morphogenetic protein (BMP)-binding loop similar to that in fetuin and the BMP receptor II, and is thus defined as the transforming growth factor-β (TGF-β) receptor II homology-1 (TRH1) domain^15^, which was shown to bind BMP^16^. The substitution p.Gly97Arg is located within the first disulfide bonded loop of Spp-24, which has not been previously characterized but would likely have important biological properties given that it’s the characteristic motif of spp-24 proteins. In general, a disulfide bond plays an important role in the folding and stability of proteins, particularly for secreted proteins^17^. Hydrophobic residues are often condensed around disulfide bond via hydrophobic interactions to form a hydrophobic core of the protein. Because the p.Gly97Arg is a switch from a hydrophobic residue to a highly hydrophilic residue that is outside of the disulfide bonded loop (Fig. 2C), this mutation may destabilize the secondary and/or tertiary structure of Spp-24 due to the interference of water molecule on amide-amide hydrogen bond, and therefore lead to folding and secretory defects of the protein. Indeed, the similarly observed cellular retention of Spp-24^Gly97Arg^ and the artificial signal peptide mutant, Spp-24^Gly29Asp^, indicates that the p.Gly97Arg has caused secretory defect of Spp-24. In addition, Spp-24 is extremely sensitive to proteolysis, and the cystatin-like domain of Spp-24 is likely conformational flexible conferring the liability to proteolysis^11^,^18^. The replacement of glycine by arginine may increase the conformational rigidity of the cystatin-like domain and subsequently decrease the susceptibility of Spp-24 to proteolysis, which may also contribute to the cytoplasmic accumulation of Spp-24^Gly97Arg^. Consistently, the Spp-24^Gly97Arg^ correlated with more severe phenotypes when compared to Spp-24^Gly29Asp^, including more intracellular retention of the protein and subsequently leading to higher rates of systemic deformity and death in zebrafish.

In cellular study, we have found that the exogenous mutant Spp-24^Gly29Asp^ or Spp-24^Gly97Arg^ co-localized with the endogenous Spp-24 protein, cause cellular retention of both the exogenous and endogenous Spp-24 proteins in the ER, and further induce ER stress. A reasonable hypothesis for this phenomenon would be that in the photoreceptors of RP patients, the mutant protein generated by the mutant *SPP2* allele would interact with the wild type protein produced by the wild type *SPP2* allele, which further leads to cellular accumulation of both Spp-24 proteins and ER stress. We therefore, based on these findings, highly hypothesized that the two *SPP2* mutations may lead to the disease in a dominant negative manner. Both insufficiency of the extracellular Spp-24 protein and cellular accumulation of the irregular Spp-24 proteins would lead to the dysfunction of rod photoreceptors or neighbor cells that support photoreceptors, e.g. RPE. In support of this proposition, the best example among cystatins is cystatin C (NP_000090.1), encoded by the *CST3* gene (NG_012887.2). Cystatin C is a member of type II cystatins characteristically possessing a signal peptide and two disulfide bridges as structural characteristics^19^. Cystatin C presents in variable ocular tissues in different species^20^,^21^,^22^,^23^ and is most abundant in the RPE of the human eye^24^,^25^, similar to the expressions of Spp-24 as detected in this study. Interestingly, the variant B cystatin C, which carries a signal peptide mutation, was correlated with increased risk of age-related macular degeneration^26^. It impaired the secretory pathway of proteins in RPE cells leading to abnormal cellular retention^25^,^27^, therefore similar to the pathogenic consequences caused by Spp-24^Gly29Asp^ and Spp-24^Gly97Arg^. Further, in the retina of rd1 mouse, a model for RP, increased cystatin C was observed in the RPE, ganglion cell layer and photoreceptors suggesting the potential involvement of cystatin C accumulation in the pathogenesis of retinal degeneration^23^. In addition to cystatin C, most proteins of the cystatin superfamily inhibit cathepsins, of which aberrant function has been implicated in RPE dysfunction^28^ and photoreceptor degenerations^29^. Abnormal cellular processing of Spp-24, as a member of the cystatin superfamily, may cause altered activities of cathepsins in retinal cells. Altogether, the aforementioned existing evidence indirectly support the hypothesis that cellular retention of Spp-24 may be a burden for RPE or photoreceptors leading to retinal degeneration.

In conclusion, *SPP2* is very likely a novel RP-causing gene. According to the genetic findings and results from *in vitro* and *in vivo* studies, we have demonstrated the pathogenicity of the mutation *SPP2* p.Gly97Arg. However, more functional studies are still needed to address the pathogenic mechanism by which the *SPP2* mutation causes degeneration of rod photoreceptors. Screening for *SPP2* mutations in RP patients with unknown genetic causes are also essential to gain better insights into the disease mechanism and genotype-phenotype correlations.

## Methods

Details on the human subjects; animals; exome sequencing and bioinformatics analysis; *in silico* analyses; RT-PCR and real time PCR; plasmids construction; cell transfection; immunoblotting; immunofluorescence of *SPP2* and cell components; whole mount *in situ* hybridization in zebrafish; mRNA synthesis and zerafish manipulations; and statistics are provided in SI Materials and Methods. All the human study was approved and prospectively reviewed by Nanjing Medical University ethnical review boards in accordance to the Declaration of Helsinki. Written informed consents were signed by all participants or their statutory guardian before their participation. The animal study was in accordance with the IACUC-approved protocol and approved by the institutional committee of NanJing Medical University. All procedures were conformed to the Guide for the Care and Use of laboratory animals.

## Additional Information

**How to cite this article**: Liu, Y. *et al.* *SPP2* Mutations Cause Autosomal Dominant Retinitis Pigmentosa. *Sci. Rep.* **5**, 14867; doi: 10.1038/srep14867 (2015).

## Supplementary Material

Supplementary Table S1-S3

Supplementary Information

## Figures and Tables

**Table 1:** Clinical Features of Attainable Patients.

Patient ID	Genotype	Status	Age at last visit (Age of Onset)*	Sex					Fundus Appearance								ERG		
					BCVA (logMAR)		Refractive Error		O.D.				O.S.					VF	
									O.D.	O.S.	O.D.	O.S.	MD	OD	AA	PD				MD	OD	AA	PD	O.D.	O.S.
AD02-II:3	c.289G>C	Het	D (20)	M	0.05	0.1	NA	NA	Yes	Waxy	Yes	Yes	Yes	Waxy	Yes	Yes	Diminished	<5°	<5°
AD02-III:2	c.289G>C	Het	50 (22)	F	0.5	0.6	−1.75 DS	−2.25 DS	Yes	Waxy	Yes	Yes	Yes	Waxy	Yes	Yes	Diminished	<15°	<15°
AD02-III:3	c.289G>C	Het	47 (23)	M	0.6	0.5	−1.50 DS	−2.00 DS	Yes	Waxy	Yes	Yes	Yes	Waxy	Yes	Yes	Diminished	<15°	<15°
AD02-IV:1	c.289G>C	Het	27 (22)	M	1.0	1.0	−0.50 DS	−1.00 DS/−0.50 DC*120	NOR	NOR	NOR	NOR	NOR	NOR	NOR	NOR	Reduced	NOR	NOR

Abbreviations: Het: heterozygous; *: year(s); D: dead; M: male; F: female; BCVA: best corrected visual acuity; logMAR: logarithm of the minimum angle of resolution; O.D.: right eye; O.S.: left eye; NA: not available; MD: macular degeneration; OD: optic disk; AA: artery attenuation; PD: pigment deposits; NOR: normal; ERG: electroretinography; VF: visual field.

**Table 2:** Characteristics of the mutations.

Variation			Exon	Bioinformatics Analysis				Frequency in Controls
Nucleotide	Amino Acid	Status		SIFT	PolyPhen	CONDEL	PROVEN	
c.289G>C	p.Gly97Arg	Het	E1	Damaging	Benign	Deleterious	Deleterious	0/800
c.85G>A	p.Gly29Asp	-	E3	Damaging	PD	Deleterious	Deleterious	0/800

Abbreviations: Het: heterozygous; E: exon; PD: probably damaging.
