# Supplementary material for: SPP2 Mutations Cause Autosomal Dominant Retinitis Pigmentosa
Source: Sci Rep. 2015 Oct 13;5:14867. doi: 10.1038/srep14867 (PMC4602186; doi:10.1038/srep14867)
Supplement: Supplementary Table S1-S3 [file srep14867-s1.doc]

***SPP2* Mutations Cause Autosomal Dominant Retinitis Pigmentosa**

Yuan Liu^1,*^, Xue Chen^1,*^, Qihua Xu^1,2^, Xiang Gao^3^, Pancy O.S. Tam^4^, Kanxing Zhao^5^, Xiumei Zhang^3^, Li Jia Chen^4^, Wenshuang Jia^6^, Qingshun Zhao^6^, Douglas Vollrath^7^, Chi Pui Pang^4^, and Chen Zhao^1,8^

*These authors contributed equally to this work.

**Supplemental Data**

Supplemental Data three tables (**Table S1-S3)**

| **Table S1. Exome Capture Statistics** | | | |
| --- | --- | --- | --- |
| **Patient ID** | **AD02- II:3** | **AD02-IV:1** | **AD02-III:5** |
| Target region (bp)^(1)^ | 51543125 | 51543125 | 51543125 |
| Raw reads | 72294784 | 86418996 | 106599434 |
| Raw data yield (Mb) | 6507 | 7778 | 9594 |
| Reads mapped to genome | 64952296 | 73736037 | 96224535 |
| Reads mapped to target region^(2)^ | 45969535 | 56083544 | 51324359 |
| Data mapped to target region (Mb) | 3438.87 | 4231.94 | 3845.2 |
| Mean depth of target region(X) | 66.72 | 82.1 | 74.6 |
| Coverage of target region (%) | 98.56 | 98.85 | 99.32 |
| Average read length (bp) | 89.94 | 89.94 | 89.93 |
| Rate of nucleotide mismatch (%) | 0.26 | 0.23 | 0.29 |
| Fraction of target covered >=4X (%) | 94.83 | 95.81 | 96.75 |
| Fraction of target covered >=10X (%) | 88.77 | 90.83 | 91.56 |
| Fraction of target covered >=20X (%) | 79.62 | 83.64 | 83.65 |
| Capture specificity (%)^(3)^ | 70.31 | 75.56 | 52.36 |
| Reads mapped to flanking region^(4)^ | 9214391 | 6364427 | 10127496 |
| Mean depth of flanking region(X) | 19.97 | 18.71 | 21.96 |
| Coverage of flanking region (%) | 94.79 | 87.59 | 97.34 |
| Fraction of flanking covered >=4X (%) | 80.23 | 64.5 | 85.11 |
| Fraction of flanking covered >=10X (%) | 56.64 | 45.3 | 60.9 |
| Fraction of flanking covered >=20X (%) | 33.39 | 29.28 | 36.4 |
| Fraction of unique mapped bases on or near target (%) | 83.39 | 83.84 | 61.89 |
| Duplication rate (%)^(5)^ | 7.16 | 12.03 | 4.86 |
| Mean depth of chrX(X) | 43.5 | 54.2 | 48.28 |
| Mean depth of chrY(X) | 81.07 | 102.53 | 84.87 |
| GC rate (%) | 43.81 | 44.67 | 43.64 |
| Gender test result | M | M | M |
| (1) Target regions here refer to the regions that are actually covered by the designed probes. (2) Reads mapped to target regions are reads that within or overlap with target region. (3) Capture specificity is defined as the percentage of uniquely mapped reads aligning to target region. (4) Flanking region refers to regions +/-200 bp on both sides of each target region. (5) PCR duplicates would have the same start and end for both mates, which rarely occur by chance. Duplication rate is the fraction of duplicated reads in raw data. | | | |

| **Table S2. Summary of Primer Information** | | |
| --- | --- | --- |
| **Gene / Plasmid** | **Forward Primer (5'→3')** | **Reverse Primer (5'→3')** |
| ***For RT-PCR in Murine Tissues*** | | |
| *Spp2 (612 bp)* | ATGGAGCAGGCAATGCTGAA | TCACTCAAAGCCAGAATTTACTCTTG |
| *β-actin (285 bp)* | GAGACCTTCAACACCCCAGC | ATGTCACGCACGATTTCCC |
| ***For RT-PCR in Human Cell Lines*** | | |
| *SPP2 (633 bp)* | ATGATTTCCAGAATGGAGAAGATGAC | TTACTCAAAGTCAGTATTTATTCTTGCTCTG |
| *β-actin (335 bp)* | GCTCGTCGTCGACAACGGCTC | CAAACATGATCTGGGTCATCTTCTC |
| ***For Exome Sequencing Analysis*** | | |
| *SPP2* | GAAAGGCACGGAACTAGGTG | CAATGTTCAGCCACGGAGTA |
| *SCRN2* | GTCCCGGACAGACTCATCT | GGGATTGCTCCACTTTACCA |
| *MLL5* | TCAATTCTTTACCAGGTCTCAC | GGGGTCTCAAAGTATCTTACAA |
| *PRIC285* | CCATGCTTCTCACACAGG | CTTCTTGACCTTCACTGTGG |
| *LRP5* | CGGACCAGACGAGGTTTGCATGTG | CCTGAATGTGCCTCAGGTTT |
| *SSTR3* | GAGCCGGCTTCATCATCTAC | CTCCTCCTCATCCTCCTCCT |
| *ANXA1* | TGGTGAGAGATGAGTTAGGAAG | AAACCACAATGTCTATCTACCC |
| *SERPINB12* | CGAGGGCTGATCTTACTGGA | GAGAGCAGACCCTGCCATAA |
| *GAD1* | CATCTTCTCTGATTGTGTCTCC | TGGGTTCAGAGAGAATAATGAC |
| *EFCAB6* | CGCATCTTTGTGACGAGTTC | GTGGACCCAGGACTGGATAA |
| *CTNND2* | GCAAATGTGATAGCACTCTGCTT | GAACTGGCCATACCTTCCAG |
| *RTN3* | CACTGGCCTTACAATACTACATGG | CCATTTGGGTTCTTGGTCTC |
| *SNRK* | GGCCAATAAGCCACAAACTG | CCAACTCAGGGAGGTCATCT |
| *TNFAIP3* | ACCAGCGTTCCAAGTCAGAT | TGACAGTTCTGCCTGACTGC |
| *C14orf183* | CCAAGAACCACACTGAATGC | CAAGAGCATGAATGGCAAGA |
| *FAT3* | CTGTCACCATCACCGTTCTG | CTGAACTCCTGGACTCACTCG |
| *MCAT* | GGTCACATGTCCACATCTAGCA | AGGCCTGCATGTTACAGCTC |
| *PIWIL3* | TGAGCTGATACCCACATGTCC | ACGAGGTAAGCCAGCTTGTG |
| *PHF21B* | GTTGGAGGCCTCTGTGGTTA | CCTCCTGCGAGGTGGTTTAT |
| *C19orf56* | GAGCCTGTCGCAGGTACAAG | CCCATCAGCAATGACAAGAC |
| *IGHV1OR21-1* | CTTGAGTGGATGGGATTGGT | TGTCCTCAGTTACTTGACCCATT |
| *FRG1B* | CTGTGCTGGTAGTTGCATGA | GGCCTAAGTGAATGAATGTCC |
| *REV3L* | TACTAGTGTTCCTCAACCCATC | TACCACTGTATCATCCCTTCTC |
| *NSMAF* | ACAGGACACACACTGGTAAAGT | AACAGATGGCTGTCTTAATGTC |
| *OR9G4* | GCAGCCGATTCTATTTGGAG | AATGGTGGTGCATCACAGAA |
| *SRRM2* | CACTAAGACAACCTCAAGGAGA | TTCTTCGAGTTACTGGAGATGT |
| *C20orf85* | AGGTGCTCAGCATCAGAGGT | CGGGAACCTTCTGGTTTAGG |
| *FYCO1* | CTCTGTTAAGGGCTTCATTCT | ACAGTTAGACGATACCAAGGTG |
| *XYLB* | TACGTTGTGTGAGTGTGAGAAA | AACGTAAGTCCTGTTGTAATCC |
| *PPL* | GATCTCTAAATCACACCTTTCG | GGAGAAGGTCACAGAGAAAGA |
| *ABHD4* | TGAAGTCACCTGGACCTTCC | CTCTTGTTCATCAGGCCAAT |
| *BAIAP2L2* | GGAAGAAATGGATGGAGATAG | GTAGGGGCTATTAACCTACTCA |
| *MS4A12* | CCTACATCTAGGCTGCATACAGAA | GCCTCGGTCTATCTGTAGAGG |
| *BRMS1* | GAGAAGCAGACACCAAGAACT | TTAGGATGGTCTAGATTTGAGG |
| *PLA2G6* | CCCTTAAGGTCTATCTCTACCC | GGAAGGATGATAGAAGTGTGTG |
| *RALGAPB* | GTGATTTTCCATGACCTGATAC | ACTATCACACTTTCCCACAAAC |
| *NCOA2* | TTATCCAATCACCTGAGACTTC | GTACAACTGGACAAGCAGAGAG |
| ***For SPP2 Coding Exon Screening*** | | |
| *Exon 1/2* | TGTCCTCAAACTGGAACCTTT | GGTTTGGATAGCCTCATGACA |
| *Exon 3* | GAAAGGCACGGAACTAGGTG | CAATGTTCAGCCACGGAGTA |
| *Exon 4* | TGTTTCTGCTTCGGTCTTGA | CCCAGGTGATGATCCAGCTA |
| *Exon 5* | CAGAGTCTTCCAGATGACATTTACA | GGGCCATCAAATGGAATTAT |
| *Exon 6* | GGAAGGGCTCTCCTTCTTTCT | CAGAGATTTAACTCCTATGAAACAGG |
| *Exon 7* | TGGGTTCAGGTTATGCTTGTC | GCAGATATTTGAAGGTCAGCAG |
| ***For Plasmid Construction*** | | |
| pCMV-C-Flag plasmid | CGCCGC**GGATCC**ATGATTTCCAGAATGGAGAAGATGAC | TGCTGC**TCTAGA**CTCAAAGTCAGTATTTATTCTTGCTCTG |
|  | *BamHI* | *XbaI* |
| pxT7 plasmid | CCGCCG**CTCGAG**ATGATTTCCAGAATGGAGAAGATGAC | CGCCGC**GGATCC**CGCTCAAAGTCAGTATTTATTCTTGCTCTG |
|  | *XhoI* | *BamHI* |

| **Table S3. Antibodies Used in This Study** | | | |
| --- | --- | --- | --- |
| **Antibodies** | **Host** | **Dilution and Application** | **Supplier** |
| Flag | Mouse | 1:500, IF; 1:2000, WB | Sigma |
| Spp-24 | Goat | 1:200, IF | Santa Cruz |
| Calnexin | Rabbit | 1:200, IF | Cell Signaling Technology |
| BiP | Rabbit | 1:2000, WB | Cell Signaling Technology |
| Ero1-Lα | Rabbit | 1:2000, WB | Cell Signaling Technology |
| IRE1α | Rabbit | 1:2000, WB | Cell Signaling Technology |
| PDI | Rabbit | 1:2000, WB | Cell Signaling Technology |
| GAPDH | Rabbit | 1:2000, WB | Bioworld |
| rhodopsin | Mouse | 1:250, IF | Abcam |
| ZPR-1  PNA | Mouse | 1:250, IF  1:200, IF | ZRIC  Life Technologies |
| Abbrevations: IF: Immunofluorescence; WB: Western Blot | | | |
