# Supplementary material for: SPP2 Mutations Cause Autosomal Dominant Retinitis Pigmentosa
Source: Sci Rep. 2015 Oct 13;5:14867. doi: 10.1038/srep14867 (PMC4602186; doi:10.1038/srep14867)
Supplement: Supplementary Information [file srep14867-s2.doc]

***SPP2* Mutations Cause Autosomal Dominant Retinitis Pigmentosa**

Yuan Liu^1,*^, Xue Chen^1,*^, Qihua Xu^1,2^, Xiang Gao^3^, Pancy O.S. Tam^4^, Kanxing Zhao^5^, Xiumei Zhang^3^, Li Jia Chen^4^, Wenshuang Jia^6^, Qingshun Zhao^6^, Douglas Vollrath^7^, Chi Pui Pang^4^, and Chen Zhao^1,8^

*These authors contributed equally to this work.

**SI Materials and Methods**

**Human subjects**

A Chinese family (**Figure 1A**) affected with adRP was recruited from the First Affiliated Hospital of Nanjing Medical University (NJMU) and was numbered as AD02. All four attainable patients and six unaffected family members participated in the present study for detailed clinical and genetic investigations. Additionally, 400 unrelated Chinese normal controls were included for mutation screening. Routine ophthalmic examinations were performed on each patient included as described previously,[^1^](#_ENREF_1) with optical coherence tomography (OCT) on those having macular involvements. Peripheral blood samples were obtained from all above mentioned participants. Genomic DNA was extracted from leucocytes in all using a QIAamp DNA Blood Mini Kit (Qiagen, Hilden, Germany) per the manufacturer’s instructions. Written informed consents were obtained from all individuals or their legal guardians before their participations. The human study was approved and prospectively reviewed by local institutional ethnical review boards in adherence to the Declaration of Helsinki.

**Animals**

C57BL/6 mice and Tuebingen zebrafish were housed in the Model Animal Research Center (MARC), Nanjing University, in accordance with the IACUC-approved protocol. Embryos were obtained from natural spawning. All procedures were conformed to the Guide for the Care and Use of laboratory animals.

**Targeted next generation sequencing, exome sequencing and bioinformatics analysis**

Targeted next generation sequencing (NGS) and exome sequencing was employed on two patients (AD02-II:3 and AD02-IV:1) and an unaffected control (AD02-III:5) from family AD02 to identify the disease-casing mutation. Six μg genomic DNA sample for each individual was fragmented into 150 to 200 bp in size, ligated with adapters to both ends, purified by the AgencourtAMPure SPRI beads, and amplified by ligation-mediated PCR (LM-PCR). A previously described microarray targeting 179 inherited retinal dystrophies (IRDs) relevant genes and 10 additional candidate genes were applied for targeted NGS approach,[^2^](#_ENREF_2) and exome sequencing was performed using the SureSelect Human All Exon v2 Kit (Agilent Technologies, Santa Clara, CA, USA) covering over 44 Mb of the human genome was then employed for enrichment of 18134 genes including approximately 187000 exons corresponding to the Consensus Coding DNA Sequence Database(CCDS). Only hybridized fragments will be captured and subjected to Agilent 2100 Bioanalyzer to evaluate the magnitude of enrichment. High-throughput next generation sequencing (NGS) was next performed on each captured library via Hiseq 2000 platform (Illumina, San Diego, CA, USA). Illumina base calling Software 1.7 was further applied to turn raw image files into 90 bp pair-end reads. Bioinformatics analyses and mutation validation was performed as detailed previously. Briefly, all detected variants were initially filtered against five SNP databases, including dbSNP137 (http://hgdownload.cse.ucsc.edu/goldenPath/hg19/database/snp137.txt.gz.), HapMap project (ftp://ftp.ncbi.nlm.nih.gov/hapmap), 1000 Genome Project (ftp://ftp.1000genomes.ebi.ac.uk/vol1/ftp), YH database (http://yh.genomics.org.cn/), and Exome Variant Server (<http://evs.gs.washington.edu/EVS/>). Noncoding variants were subsequently removed, and we focused on variants located within exons and flanking 10 bp of intronic region. These variants were further filtered against our in-house ES database derived from 1400 individuals unrelated to RP provided by BGI-Shenzhen.

***In silico* analyses**

Evolutionary conservation of the mutated amino acids was obtained using Vector NTI Advance™ 2011 (Invitrogen, Carlsbad, CA, USA) by aligning the protein sequence of human Spp-24 (NP_008875.1) with those of the following orthologous Spp-24 proteins derived from GenBank or Ensembl Genome Browser: *Pan troglodytes* (XP_001151806.1), *Canis lupus familiaris* (XP_852985.2), *Bos taurus* (NP_776613.2), *Sus scrofa* (ENSSSCP00000017287), *Rattus norvegicus* (NP_446029.1), *Gallus gallus* (ENSGALP00000039012 ),and *Danio rerio* (ENSDARP00000115588). Crystal structural modeling of the wild type (WT) and two mutant Spp-24 proteins were built from SWISS-MODEL online server, with the predicted structures demonstrated via PyMol software (version 1.5). Four online tools were applied to determine the effects of the missense substitutions namely SIFT (<http://sift.jcvi.org/>),[^6^](#_ENREF_6) PolyPhen-2 (v.2.2.2; <http://genetics.bwh.harvard.edu/pph2/>),[^7^](#_ENREF_7) CONDEL (http://bg.upf.edu/condel/analysis),[^8^](#_ENREF_8) and PROVEAN (v.1.1.3; http://provean.jcvi.org/index.php).[^9^](#_ENREF_9) For amino acid changes located in signal peptide, an additional online prediction tools were applied called SignalP (version 4.1; http://www.cbs.dtu.dk/services/SignalP/).[^10^](#_ENREF_10)

**RT-PCR**

To visualize the expression levels of the *Spp2* transcript in multiple murine tissues, we performed reverse-transcriptase polymerase chain reaction (RT-PCR) on the cDNA templates generated from diverse tissues of C57BL/6 mice, including heart, liver, brain, lung, kidney, intestines, stomach, spleen, muscle, neural retina, retinal pigment epithelium (RPE), and optic nerve. Two human cell lines called human retinal pigment epithelium (ARPE19) and human embryonic kidney (HEK) 293T were also included for expression analysis using RT-PCR. Total RNA from multiple murine tissues and two human cell lines was extracted using Trizol (Invitrogen), and cDNA was further synthesized with a reverse-transcription kit (Invitrogen). Subsequently, semi-quantitative PCR was conducted according to a previous protocol. All generated products were confirmed by Sanger sequencing. Primer information and size of products were detailed in **Supplemental Table 2**.

**Plasmids construction**

The full open reading frame (ORF) sequence of human *SPP2* (NM_006944.2) was synthetized and used as a template for amplification. Specific primers matching the *SPP2* sequence at their 3’ moiety and restriction sites for endonucleases at their 5’ moiety as detailed in **Supplemental Table 2** were used to clone the WT full-length *SPP2* into the Flag-tagged vector pCMV-C-Flag (Beyotime, Nanjing, China) to generate the recombinant plasmid AcFlag-Spp-24^WT^ for transfection assay, and pxT7 plasmid (a gift from Dr. Anming Meng, Tsinghua University) to produce AcpxT7-Spp-24^WT^ for *in vivo* transcription assay. Using a QuikChange lightning site-directed mutagenesis kit (Agilent Technologies), the two missense mutations identified in this study, c.G85A and c.G289C, were further introduced into the generated WT plasmids to obtain recombinant plasmids including AcFlag-Spp-24^Gly29Asp^, AcFlag-Spp-24^Gly97Arg^, AcpxT7-Spp-24^Gly29Asp^, and AcpxT7-Spp-24^Gly97Arg^. The inserted sequences of all produced plasmids were validated via Sanger sequencing in both directions.

**Cell transfection**

HEK 293T cells were maintained in Dulbecco’s modified Eagle’s medium (DMEM) supplemented with 10% fetal bovine serum (FBS; Invitrogen, Carlsbad, CA, USA) at 37°C, 5% CO_2_. For immunoblotting assay, cells were seeded into 6-well templates, and for immunofluorescence analysis, cells grew in 8-well chamber slides (Millipore, Billerica, MA, USA). At 50% to 60% confluence, cells were transfected withAcFlag-Spp-24^WT^ or AcFlag-Spp-24^Gly29Asp^ or AcFlag-Spp-24^Gly97Arg^, using Lipofectamin^TM^ 2000 Transfection reagent (Invitrogen) per the manufacturer’s protocol. At 24 hours post transfection, cells were harvested and prepared for immunoblotting or immunofluorescence analyses. The culture medium was also collected at 24 hours post transfection for enzyme-linked immuno sorbent assay (ELISA).

**Immunoblotting and ELISA**

Proteins were extracted from cells and were resolved by 10% SDS-polyacrylamide gel electrophoresis (SDS PAGE) and transferred to a polyvinylidene fluoride membrane (Millipore, Billerica, MA, USA). The membrane was blocked for 1 hour at room temperature with 5% skim milk in Tris-buffered saline containing 0.05% Tween 20 (TBST), incubated with the anti-Flag, anti-BiP, anti- Ero1-Lα, anti- IRE1α, and anti- PDI polyclonal antibodies (**Supplemental Table 3**) overnight at 4°C, washed with TBST for 5 times, and probed with peroxidase-conjugated goat anti-mouse antibody (**Supplemental Table 3**) for 1 hour at room temperature. After another 5 times wash, the blots were developed by autoradiography with the ECL-Western blotting system (Amersham Bioscience, NJ, USA) per the manufacturers’ protocols. The collected culture medium were used to assess the expression level of secreted Spp-24 using a commercial human SPP2/SPP-24 ELISA kit (CUSABIO, Wuhan, China) per the manufacturer’s protocol.

**Whole mount *in situ* hybridization in zebrafish**

Full-length cDNA of *spp2*, the analogous of human *SPP2* in zebrafish, was cloned as template for generation of the dioxigenin-labeled anti-sense RNA probes per manufacturer’s directions (Roche Applied Science, Mannheim, Germany). Whole mount *in situ* hybridization in zebrafish was performed according to the modified standard protocols as detailed previously. Briefly, embryos for observation after 24 hours post fertilization (hpf) was initially treated with 0.003% 1-phenyl-2-thiourea (Sigma, MA, USA) from 8 hpf to reduce melanin pigmentation, and further incubated with proteinase K (Roche) for penetration of RNA probes into the embryos. All embryos were mounted in a mixture of benzylbenzoate and benzylalcohol (2:1) for 4 hours at room temperature to develop hybridization signals. A Leica MZ16 FA Fluorescence Stereomicroscope (Leica) was then used for photomicrographs collecting.

**mRNA synthesis and zebrafish manipulations**

Human WT *SPP2* mRNA (Spp-24^WT^) and mutant mRNAs carrying p.Gly29Asp (Spp-24^Gly29Asp^) and p.Gly97Arg (Spp-24^Gly97Arg^) were synthesized from the above mentioned linearized plasmids AcpxT7-Spp-24^WT^, AcpxT7-Spp-24^Gly29Asp^and AcpxT7-Spp-24^Gly97Arg^, respectively, using the mMESSAGE mMACHINE T7 Ultra Kit (Ambion, USA) in accordance with the manufacturer’s instructions. In addition, antisense mRNA (Spp-24^Anti^) was also obtained from the plasmid AcpxT7-Spp-24^WT^ with a reversed transcriptional direction. The generated mRNAs were subsequently purified with the RNeasy Kit (Qiagen, Hilden, Germany). Purified mRNAs were injected into the 1- to 2-cell-stage (0 day post fertilization [dpf]) zebrafish embryos. A total of 100 pg (1 nl) mRNAs was injected into zebrafish embryos, which were separated into four mRNA-injected groups, including Spp-24^Anti^ (n = 64), Spp-24^WT^ (n = 72), Spp-24^Gly29Asp^ (n = 68), and Spp-24^Gly97Arg^ (n = 70). The counting and the percentage calculation of zebrafish embryos were conducted from 2 dpf to 4 dpf as detailed previously.[^2^](#_ENREF_2) Briefly, for all injections, zebrafish dead within 24 h after injection were excluded as such death was likely resulted from unspecific causes. The deformation and death of zebrafish were counted from 2 dpf to 4 dpf. At 4 dpf, zebrafish with relatively normal appearance were collected from each injected group.

**Q-PCR analyses**

Q-PCR was performed for the detection of the exogenous and endogenous *SPP2* expression in different groups of zebrafish injected with Spp-24^Anti^, Spp-24^WT^, Spp-24^Gly29Asp^ and Spp-24^Gly97Arg^, respectively, using FastStart Universal SYBR Green Master (ROX; Roche, Basel, Switzerland) with the StepOne Plus Real-time PCR System (Applied Biosystems, Darmstadt, Germany) according to the manufacturer’s instructions.

**Immunofluorescence**

We performed immunofluorescence to determine the expression distribution of Spp-24 in mouse retina, to visualize the subcellular localization of the endogenous Spp-24 and Flag-tagged exogenous Spp-24 in transiently transfected HEK 293T cells, and to determine the morphology of the rod and cone photoreceptor cells in zebrafish from different injected groups. For expression analysis, the eye cup of an 8-week-old mouse was obtained per standard procedures. For frozen section, the eye cup was fixed in 4% paraformaldehyde (PFA) at 4 °C overnight, incubated with 30% sucrose for 2 hours, embedded with optimal cutting temperature solution, and frozen in liquid nitrogen for 1 minute. A Lecia CM1900 cryostat (Leica, Wetzlar, Germany) was applied for sectioning at 5 µm. Immunofluorescence was further conducted using a previously described protocol.[^2^](#_ENREF_2) Cryosections were incubated with [Spp-24 (+)] or without [Spp-24 (-)] anti-mouse spp-24 primary antibody, then treated with fluorescence-conjugated secondary antibodies (Invitrogen), and finally counterstained by 4’, 6-diamidino-2-phenylindole (DAPI; Sigma, USA). Images were taken using an Olympus IX70 confocal laser-scanning microscope (Olympus, Tokyo, Japan). For cellular study, harvested HEK 293T cells were incubated with anti-Flag/anti-Spp-24/anti-Calnexin primary antibody, and in zebrafish study, anti-zebrafish rhodopsin antibodies, anti-zebrafish ZPR-1 antibodies, and peanut agglutinin (PNA) lectin were used to label rod inner/outer segments (IS/OS), red/green double cones,and cone OS, respectively. Information of the antibodies was detailed in **Supplemental Table 3**.

**Statistics**

We used GraphPad Prism (version 4.0; GraphPad Software, San Diego, CA, USA) for statistical analysis. One-way ANOVA or Student’s T-test was applied for comparisons between different groups. Data was presented as mean ± standard deviation (SD), and P < 0.05 was taken as statistically significant.

**Reference**

1. Zhao, C. *et al.* A novel locus (RP33) for autosomal dominant retinitis pigmentosa mapping to chromosomal region 2cen-q12.1. *Hum Genet***119**, 617-23 (2006).

2. Chen, X. *et al.* PRPF4 Mutations Cause Autosomal Dominant Retinitis Pigmentosa. *Hum Mol Genet* (2014).

3. Chen, X. *et al.* Targeted sequencing of 179 genes associated with hereditary retinal dystrophies and 10 candidate genes identifies novel and known mutations in patients with various retinal diseases. *Invest Ophthalmol Vis Sci***54**, 2186-97 (2013).

4. Arnold, K., Bordoli, L., Kopp, J. & Schwede, T. The SWISS-MODEL workspace: a web-based environment for protein structure homology modelling. *Bioinformatics***22**, 195-201 (2006).

5. Kiefer, F., Arnold, K., Kunzli, M., Bordoli, L. & Schwede, T. The SWISS-MODEL Repository and associated resources. *Nucleic Acids Res***37**, D387-92 (2009).

6. Kumar, P., Henikoff, S. & Ng, P.C. Predicting the effects of coding non-synonymous variants on protein function using the SIFT algorithm. *Nat Protoc***4**, 1073-81 (2009).

7. Adzhubei, I.A. *et al.* A method and server for predicting damaging missense mutations. *Nat Methods***7**, 248-9 (2010).

8. Gonzalez-Perez, A. & Lopez-Bigas, N. Improving the assessment of the outcome of nonsynonymous SNVs with a consensus deleteriousness score, Condel. *Am J Hum Genet***88**, 440-9 (2011).

9. Choi, Y., Sims, G.E., Murphy, S., Miller, J.R. & Chan, A.P. Predicting the functional effect of amino acid substitutions and indels. *PLoS One***7**, e46688 (2012).

10. Petersen, T.N., Brunak, S., von Heijne, G. & Nielsen, H. SignalP 4.0: discriminating signal peptides from transmembrane regions. *Nat Methods***8**, 785-6 (2011).

11. Zhao, C. *et al.* Autosomal-dominant retinitis pigmentosa caused by a mutation in SNRNP200, a gene required for unwinding of U4/U6 snRNAs. *Am J Hum Genet***85**, 617-27 (2009).

12. Liang, D. *et al.* Expressions of Raldh3 and Raldh4 during zebrafish early development. *Gene Expr Patterns***8**, 248-53 (2008).

13. Zhao, Q., Dobbs-McAuliffe, B. & Linney, E. Expression of cyp26b1 during zebrafish early development. *Gene Expr Patterns***5**, 363-9 (2005).
